# Remodeling of the lower and upper airways

**DOI:** 10.1016/S1808-8694(15)30847-8

**Published:** 2015-10-18

**Authors:** Guilherme de Toledo Leme Constantino, João Ferreira de Mello

**Affiliations:** 1 Otorhinolaryngologist. Fellow in endonasal endoscopic surgery, HC/FMUSP.; 2 Livre-Docente (habilitation) professor of the Otorhinolaryngology Discipline, Faculdade de Medicina da Universidade de Sao Paulo. Hospital das Clínicas, Faculdade de Medicina da USP/Sao Paulo.

**Keywords:** asthma, remodeling, rhinitis

## Abstract

Remodeling is defined as modeling again or differently, as reconstructing. Remodeling is a critical aspect of wound repair in all organs; it represents a dynamic process that associates the production and degradation of matrix in reaction to inflammation. This leads to normal reconstruction or a pathologic process. **Aim and Methods:** To compare data in the current literature on upper and lower airways. **Results:** Asthma is a chronic inflammatory disease associated with abnormal airways remodeling. In allergic rhinitis, another chronic inflammatory disease, remodeling is still poorly understood. Even though inflammation is similar in allergic rhinitis and asthma, the pathologic extent of nasal remodeling, as well as its clinical consequences, might be different from those in bronchi. **Conclusion:** Remodeling occurs less in upper airways compared to lower airways; it is apparent, however, that the structure of the rhinitic nose is not normal.

## INTRODUCTION

Remodeling may be defined as modeling again or rebuilding. It is a critical aspect of injury repair processes in every organ of the body; either normal tissue is rebuilt or pathological tissue is formed.^1^ The main histological features of chronic inflammation and remodeling are: macrophage and lymphocyte infiltration, fibroblast proliferation (which may become myofibroblasts), angiogenesis, increased connective tissue (fibrosis) and tissue destruction.^2^

Allergic rhinitis is inflammation of upper airways; inflammation alone, however, does not explain the chronic nature of this condition. The role of the bronchial epithelium as a key regulator of airway inflammation and remodeling responses may be demonstrated in asthma. Many studies are trying to demonstrate the same finding in upper airways.^3^

Although asthma may be seen as a reversible airflow obstruction condition, many adult and child asthmatics show evidence of residual airway obstruction, which is detected even in asymptomatic patients. This irreversible component of airway obstruction results from structural bronchial alterations that change the shape of airways and result in variable degrees of loss of function.^2^, ^4^

The purpose of this paper was to compare existing data in the literature on upper and lower airway remodeling, since there are few published papers in our setting on this theme.

## REVIEW OF THE LITERATURE

### 1 Lower airways

Airway remodeling is a dynamic process in which extracellular matrix is deposited and degraded in response to trauma, resulting in reconstruction of damaged tissue that is very important for healing.^2^ Remodeling in asthma has been studied in detail; it includes changes in airway epithelium, lamina propria and submucosa, in which the walls become thickened. Inflammation in asthma may be mediated by Th^2^ lymphocytes, which secrete cytokines that orchestrate cell inflammation and bronchial hyper-responsiveness.^5^

#### 1.1 Histopathological features

##### Thickening of the reticular basal membrane

The epithelial basement membrane in human airways is composed of two layers, the basal lamina, which is considered the true basement membrane and which is not thickened in asthmatics, and the reticular lamina, which is formed by types I, III and V, and fibronectin, and is thickened is asthma.^6^ This is a common, consistent and early feature in the pathology of asthma.^7^ There is plexiform deposition of immunoglobulins, collagen I and III, tenascin and fibronectin; there is no evidence of laminin deposition.^2^, ^8^ These proteins are produced by activated myofibroblasts, which leads to subepithelial fibrosis.^2^, ^9^ Thickening of the basement membrane was not related to the severity, duration or etiology of asthma in some studies;^10^ others, however, have reported a correlation between these findings and disease severity.^11^

##### Interstitial matrix

The presence of an abnormal superficial network of elastic fibers has been reported in asthmatics, which suggests an elastolytic process. Deeper elastic fibers are also altered in many asthmatics.^2^, ^12^

The submucosa of asthmatics may show collagen fiber hyperplasia, and fibronectin, laminin and tenascin deposits.^2^, ^13^

##### Blood vessels

Increased vasculature, vasodilatation and microvascular extravasation are features of airway wall remodeling. Patients with fatal asthma have an increased number of blood vessels; in these cases, however, the extension of neovascularization and angiogenesis remain uncertain.^14^, ^15^

##### Smooth muscle

Studies on cadavers have shown bronchial smooth muscle hypertrophy and hyperplasia in asthmatics.^16^ Animal experiments have shown that prolonged exposure to allergens may increase the thickness of airway smooth muscles.^17^

##### Glands

Remodeling in asthma may include goblet cell hyperplasia, mucous gland hypertrophy and increased mucus production.^18^, ^19^

#### 1.2 Mediators

CD4+ lymphocytes have an important role in remodeling responses; they generate Th2 cytokines, such as interleukins (IL) 5 and 13, and promote fibrosis.^6^, ^20^, ^21^ Eosinophils are highly active in asthma remodeling, particularly through the transforming growth factor β1 (TGF-β1), one of its main mediators.^20^

Other mediators with elevated levels are: the epidermal growth factor (EGF), the fibroblast growth factor, the vascular endothelial growth factor (VEGF), IL-11, elastase, endothelin, matrix metalloproteinases (MMPs) 2 and 9, and the tissue inhibitor of metalloproteinase 1 (TIMP-1).^2^, ^22^, ^23^

Mast cells are also important in remodeling; they activate mesenchymal cells by mediators such as tryptase, a potent stimulator of fibroblast and smooth muscle cell proliferation.^24^, ^25^ Another mediator released by mast cells is the type 1 plasminogen activation inhibitor, which is also associated with remodeling.^26^

The role of certain genes is being debated, such as the anchored protein gene containing a disintegrin and a metalloproteinase on domain 33 gene (ADAM 33), which may affect tissue repair processes.^23^, ^27^

#### 1.3 Response to therapy

There has been much about the possibility of preventing remodeling in asthma by early treatment with various drugs. Inhaled corticosteroids are the mainstay of asthma therapy. It is thought that the anti-inflammatory effect of these drugs may - at least partially - reduce the amount of cytokines and mediators that are responsible for chronic inflammation and remodeling in this condition.^28^, ^29^ Inhaled corticosteroids are able to decrease subepithelial fibrosis, basement membrane thickness and bronchial hyperreactivity. Airway vascularization is also decreased by inhaled corticosteroid therapy, although VEGF levels remain unchanged.^30^, ^31^ Other drugs such as leukotriene inhibitors, ketotifen, sodium cromoglycate and teophilin are anti-inflammatory drugs, but less effective than corticosteroids. The role of these drugs in remodeling remains uncertain.^28^, ^29^, ^30^

Immune stimulators, such as anti-IL-5 and the CpG-containing immune stimulatory sequence (CpG DNA), are a future perspective. These drugs decrease TGF-β1 expression, and therefore remodeling.^20^

### 2 Upper airways

#### 2.1 Allergic rhinitis

Although inflammation is similar in allergic rhinitis and asthma, remodeling is less pronounced in rhinitis. The reasons for this are not clear, and two hypotheses have been raised: cytokines may be produced specifically by bronchial smooth muscle cells, and these conditions may have a different embryonic origin (the nose is ectodermal and bronchi are endodermal), where persistent (asthma) or re-expressed (rhinitis) embryologic differentiating genes lead to different remodeling patterns.^23^

##### 2.1.1 Histopathological features

###### Epithelium

Few studies have investigated the nasal epithelium in allergic rhinitis patients; those that have show conflicting results. These patients have signs of remodeling not only in the nasal mucosa but also in the bronchial epithelium, regardless of the presence or absence of asthma. Braunstahl et al. demonstrated that there were more eosinophils in the nasal and bronchial mucosa, a thickened basement membrane, and epithelial desquamation in the bronchial mucosa of allergic rhinitis patients with no asthma. There was, however, no structural change in the nasal mucosa.^32^ The definition of abnormality in this study was the presence of clear rupture, similar to asthma. In the latter, this is a significant finding, probably related to dynamic forces on the epithelium during bronchoconstriction, which is absent in the nose.^33^ Although there is no clear epithelial rupture in allergic rhinitis as there is in asthma, there may be subtle changes detected only by electron microscopy, such as cytoplasmatic vacuoles and increased intercellular space.^34^ Ciliated cell dysplasia and metaplasia may be found in allergic rhinitis.^35^

###### Reticular basal membrane

Similar to asthma, the reticular portion of the basement membrane is thickened in the nasal epithelium of allergic rhinitis patients, as has been shown in a number of papers.

Chakir et al. biopsied the bronchi of non-asthmatic allergic rhinitis patients and found that type I and III collagen and fibronectin were elevated in the reticular portion of the basement membrane; this was associated with a network of myofibroblasts along the epithelium, similar to findings in asthma, albeit less intense. These authors concluded that subepithelial fibrosis in rhinitis results from the deposition of type I and III collagen and fibronectin, which are produced by bronchial myofibroblasts.^36^

Montero et al. biopsied the lower turbinate in 26 untreated allergic rhinitis patients and found that the basement membrane was thickened in 92.3% of cases, that there was subepithelial fibrosis in 92.4% of cases, neutrophils, eosinophils and lymphocytes were found in 100% of cases, edema in 46.2% of cases and vascular dilatation was present in 11.1% of cases, suggesting that remodeling was taking place in allergic rhinitis.^37^ Sanai et al. compared the mucosa of the lower turbinate in allergic rhinitis patients with non-allergic controls and found that type I and III collagen was much more significantly deposited in the reticular portion of the basement membrane with resulting thickening in allergic rhinitis. The total amount of collagen, however, was similar in both groups; the authors suggested that nasal mucosa remodeling occurs immediately below the epithelium in the basement membrane, by collagen deposition.^38^ Agha-Mir-Salim et al. studied the basement membrane of the lower turbinate and found that thickening of this layer is common, as it helps stabilize the epithelium mechanically. Collage deposits, therefore, would act as a mechanical control mechanism to avoid excessive expansion of the nasal mucosa, thus keeping the nasal cavity patent.^39^ Sanai et al. have suggested that allergic inflammation accelerates the physiological mechanism of collagen deposition in the basement membrane of the lower turbinate, resulting in fibrosis.^38^

###### Glands

The nasal mucosa contains goblet cells, which are unicellular mucus-secreting glands located above the epithelial basement membrane; there are also deep glands in the lamina propria. There is no consensus about whether goblet cells are more numerous in rhinitis; there are, however, structural changes such as distended acini with secretion, and acini and duct degeneration and obstruction. Compression, atrophy and dilatation may be found in deep mucosal glands.^33^

###### Blood vessels

Angiogenesis has not been clearly established in allergic rhinitis. A study measuring vessel surface and volume density has shown no difference between normal and rhinitis patients.^40^ On the other hand, angiogenic factors such as the platelet-derived endothelial cell growth factor and the VEGF have been found at elevated levels in rhinitis patients.^23^, ^33^ Other indicators of vascular change may be seen, such as arteriolar wall thinning and destruction, globulin and complement deposits on vascular walls and vasodilatation.^33^

##### 2.1.2 Mediators

Remodeling, collagen deposits and other extracellular matrix product deposition may be at least partially attributed to an increased fibroblast number and activity. Ongoing research is searching for mediators and cytokines such as the TGF-β1 and the granulocyte macrophage colony growth factor (GM-CSF), which may be released by local inflammatory cells to activate those fibroblasts.^41^, ^42^ Nitric oxide (NO) is a key mediator for the endothelium-derived relaxing factor (EDRF) that is responsible for relaxing vascular smooth muscle.^43^ NO is elevated in asthmatic and allergic rhinitis patients, and may be used in future as a marker for airway inflammation. There is also interest in NO as an inflammation and remodeling agent. Tewfik et al. demonstrated that NO stimulates collagen expression by fibroblasts derived from nasal polyps in patients tested positive for allergy.^42^

Various other cytokines have been investigated for their possible role in airway remodeling. Oncostatin M (IL-6 family), for instance, is expressed in the nasal mucosa and has been found to be elevated in allergic rhinitis patients.^44^ The heme oxygenase enzyme, an antioxydating agent that catabolizes heme into carbon monoxide (CO) and biliverdin, has an important cell-protecting action against oxidative injury. Elhini et al. found that heme oxygenase 1 (HO-1) was elevated in the tissues of allergic rhinitis patients, and concluded that improved understanding of HO-1 expression may improve the management of rhinitis in future.^45^

MMPs are proteolytic enzymes involved in extracellular matrix turnover.^46^ Studies of MMP-9 have not demonstrated any elevation in perennial allergic rhinitis;^47^ however, 3 to 10 hours after exposure to nasal allergens, the eosinophilic cationic protein (ECP) and MMP-9 are elevated.^48^ Significant amounts of TIMP-1 and TIMP-2 mRNA may be found in the nasal mucosa of perennial allergic rhinitis patients.^47^

Table 1 contains a summary of upper and lower airway remodeling.

**Table 1:** Comparison of remodeling between upper and lower airways.

	Lower airways	Upper airways
Epithelium	Evident structural changes with rupture franca	Subtle structural changes
Reticular basement membrane	Mainly thickening and collagen I and III deposits	Similar to lower airways
Blood vessels	Increased vascularization, vasodilatation and microvascular extravasation	Angiogenesis not yet demonstrated conclusively. Presence of mild vascular changes
Glands	Goblet cell and mucous gland hyperplasia and increased mucus production	Distended, degenerated and obstructed acini and ducts
Response to therapy	Inhaled corticosteroids decrease subepithelial fibrosis, there is decreased basement membrane thickening and bronchial hyperreactivity	Absence of conclusive studies

#### 2.2 Chronic rhinosinusitis

Remodeling of the mucosa is also found in chronic sinus disease, similar to asthma and allergic rhinitis. Ponikau et al. encountered increased basement membrane thickening, epithelial injury and heterogeneous eosinophilic inflammation in the sinus mucosa of allergic and non-allergic chronic rhinosinusitis (CRS) patients, suggesting that a similar process occurs in asthma and CRS.^49^ Sobol et al. compared the sinus mucosa of CRS adults and children with controls and found an increased number of eosinophils and subepithelial collagen deposits in both CRS adults and children (to a lesser degree). These results suggest that remodeling begins early and, if not interrupted, may cause irreversible changes in the sinus mucosa.^50^

##### 2.3 Response to therapy

Some allergic rhinitis and CRS patients respond very well to medical treatment, especially therapy with nasal corticosteroids. Other, however, do not benefit from such therapy, and become candidates for surgery. The issue is to find the difference between both groups and by how much remodeling is part of this difference.

## FINAL COMMENTS

Tissue remodeling in response to chronic inflammation has been own in all body systems. It has been widely demonstrated in lower airways, especially in asthmatics. Upper airway remodeling is being investigated; there is strong evidence suggesting that it occurs in allergic rhinitis and CRS, particularly by eosinophilic inflammation. Upper airway remodeling is less intense compared to lower airway remodeling; it is, however, apparent that the nasal mucosa in rhinitis patients is not normal. Further studies are needed, since many steps need to be clarified before the whole process is understood.
